# YOLOPears: a novel benchmark of YOLO object detectors for multi-class pear surface defect detection in quality grading systems

**DOI:** 10.3389/fpls.2025.1483824

**Published:** 2025-02-17

**Authors:** Junsheng Chen, Haoxuan Fu, Chuhan Lin, Xian Liu, Lijin Wang, Yaohua Lin

**Affiliations:** ^1^ Fujian Agriculture and Forestry University, Fuzhou, China; ^2^ Key Laboratory of Smart Agriculture and Forestry (Fujian Agriculture and Forestry University), Fujian Province University, Fuzhou, China; ^3^ Digital Agriculture Research Institute, Fujian Academy of Agricultural Sciences, Fuzhou, Fujian, China

**Keywords:** pear, dataset, pear surface defect detection, smart agriculture, deep learning, computer vision

## Abstract

Pears are one of the most widely consumed fruits, and their quality directly impacts consumer satisfaction. Surface defects, such as black spots and minor blemishes, are crucial indicators of pear quality, but it is still challenging to detect them due to the similarity in visual features. This study presents PearSurfaceDefects, a self-constructed dataset, containing 13,915 images across six categories, with 66,189 bounding box annotations. These images were captured using a custom-built image acquisition platform. A comprehensive novel benchmark of 27 state-of-the-art YOLO object detectors of seven versions Scaled-YOLOv4, YOLOR, YOLOv5, YOLOv6, YOLOv7, YOLOv8, and YOLOv9,has been established on the dataset. To further ensure the comprehensiveness of the evaluation, three advanced non YOLO object detection models, T-DETR, RT-DERTV2, and D-FINE, were also included. Through experiments, it was found that the detection accuracy of YOLOv4-P7 at mAP@0.5 reached 73.20%, and YOLOv5n and YOLOv6n also show great potential for real-time pear surface defect detection, and data augmentation can further improve the accuracy of pear surface defect detection. The pear surface defect detection dataset and software program code for model benchmarking in this study are both public, which will not only promote future research on pear surface defect detection and grading, but also provide valuable resources and reference for other fruit big data and similar research.

## 1 Introduction

Pear is one of the major fruits in the world, is the third economically most important fruit after apple, citrus. In 2022, global pear production reached approximately 23 million tons. China is the world’s largest pear producer, accounting for more than two-thirds of the global production. In 2022, China’s pear production reached 19,265,300 tons (USDA Foreign Agricultural Service, 2024; IndexBox, 2024). Other major producing countries include Argentina, Italy, the United States, and Turkey. As a high-value fruit, pears are popular not only for fresh consumption but also for their extensive use in processed foods. They play an important role in promoting rural economic development and increasing farmers’ income. However, the pear industry faces significant losses due to pests, surface bruising, and storage issues. For instance, Korla fragrant pears suffer up to a 30% annual loss during storage due to pathogenic infections, and a rot rate as high as 15% due to surface defects, resulting in annual economic losses exceeding 60 million RMB (IndexBox, 2024).

At present, the detection of defect types and defect severity in pears mainly relies on manual sampling or physical and chemical methods. Long-term use of eyes will cause visual fatigue, and different people have different judgment standards, resulting in high error rate and low reliability in defect recognition (Zhou et al., 2013). Therefore, finding a more efficient and accurate method to deal with pear surface defects has become an urgent problem to be solved in the current development of the pear industry.

In fruit surface defect detection, computer vision technology plays an important role. Zhou et al (Zhou et al., 2013). proposed a method to distinguish calyx, fruit stalk and defects, which uses a simple morphological method to detect defects and remove the background. The experimental results showed the universality and accuracy in successfully detecting three pear surface defects. Chen (Chen, 2021). highlighted the original features and defect features of Korla fragrant pear respectively by greying the image and image enhancement, reducing the redundant image information. A recognition algorithm to distinguish the outer contour and defective region of Korla fragrant pear was investigated by combining the improved threshold segmentation of bimodal thresholding method, edge detection and morphological processing. Ireri (Ireri et al., 2019). developed a tomato grading machine vision system based on RGB, which detects the calyx and stalk scar of normal and defective tomato based on the histogram thresholding respectively, and the accuracy of the recognition was 95.15%. These computer vision techniques can also identify the surface defects of pears significantly.

There are some fruit sorters commercially available, grading fruits only based on their color and size in the image. This kind of sorters is too crude to detect fruits with surface defects. Hu (Hu, 2022). combined visible/near-infrared spectroscopy detection technology and embedded systems to develop a portable pear disease detection device, which can detect typical diseases of pear fruits in the outdoor or detection site. At present, the actual performance of this technology still needs to be fully verified before it can be widely promoted and adopted. Furthermore, in the field of fruit surface defect detection, traditional object detection methods based on image processing and machine learning require manual extraction of features (color, texture, morphology, etc.), which is inefficient and only applicable to specific objects.

At the same time, a lot of research has been carried out on image processing image/analysis techniques for pear surface defect detection (Chen et al., 2021; Hu, 2022). The geometric, color and texture features of defects have been proposed to be studied and analyzed, and combined with decision trees to construct classification algorithms for identifying defects in pears. Although easy to compute, most of them are not robust to various imaging conditions, but they can only classify pear images at the pixel level and cannot accurately locate the position of defects (rectangular boxes). In recent years, pear surface defect detection has started to use data-driven deep learning (DL) based algorithms. Satisfactory classification or detection accuracy (Xie et al., 2023; Sun et al., 2022; Zhang et al., 2021) can be achieved by using well-trained models with a large number of datasets.Trained DL models can be deployed on computational hardware (e.g., NVIDIA Jetson AGX Xavier module), which is suitable for pear classification platforms to realize real-time pear defects and classifications.

Fruit defect detection tasks can be divided into three basic categories: 1) classifying images as defective or defect-free, 2) detecting or localizing defective regions in an image, and 3) segmenting images into semantic surface defect maps. This corresponds to the three basic problems of computer vision - image classification, target detection and semantic segmentation. Deep learning methods are commonly used to train feature surface error classification models based on image-level labeled datasets (Sun et al., 2022; Zhang et al., 2021).However, the resulting model does not provide information about the location of specific defects in the image, and is therefore imperfect for tasks that require obtaining a precise localization of defects for grading purposes. In contrast, object detection that requires localizing objects of interest within an image (Girshick et al., 2015), which predicts the specific location of defects in an image, is more conducive to highly accurate of fruit quality grading.

There are two main types of deep learning object detectors (Liu et al., 2020), the first is a two-stage detector that generates object proposal in the first stage, and then performs object categorization and bounding box regression on these proposal in the second stage. The other is single-stage detector which is end-to-end and does not require region proposals process, so single-stage detector has higher computational efficiency and faster inference. While R-CNN (Girshick et al., 2015), Faster-RCNN (Ren et al., 2016) and Mask-RCNN (He et al., 2017) these two-stage detectors are not as suitable as the single segment detectors for real-time applications, especially on embedded devices with resource constraints. YOLO is the most familiar single-segment detector, which was originally proposed by Redmon et al (Redmon et al., 2016), and then the original authors upgraded it twice as for YOLO 9000 (Redmon and Farhadi, 2017) and YOLOv3 (Redmon and Farhadi, 2018). YOLOv3 achieved a good balance between accuracy and inference speed and was widely used in the previous years. The original authors did not continue with version updates after YOLOv3. However, other researchers have modified it to continue to improve the accuracy and inference speed of the model, including YOLOv4 (Bochkovskiy et al., 2020), Scaled-YOLOv4 (Wang C. Y. et al., 2021a), and YOLOv5 (Jocher, 2022), YOLOR (Wang C. Y. et al., 2021b), YOLOv6 (Li et al., 2022) and YOLOv7 (Wang C. Y. et al., 2023), YOLOv8 (Jocher, 2023). The latest version, YOLOV9, was released on February 21, 2024 (Wang C. Y. et al., 2024). Each YOLO detector pair is available in different sizes and can be configured on demand.

The most widely used detector for detecting surface defects in fruit is the YOLO. Xin Y. et al (Xin et al., 2021). compared the performance of SVM, Fast RCNN, YOLOv2 and YOLOv3 models on apple images. The results showed that the YOLOv3 model was the most suitable for apple defect detection. Valdez et al (Valdez, 2020). considered apple defect detection as an object detection problem. Having compared the Single-Shot Detector (SSD) with YOLOv3, they trained the chosen YOLOv3 model on a dataset containing both normal and defect apples to detect which apples are normal. Liang et al (Liang et al., 2022). proposed a real-time tomato surface defect detection method based on model pruning. Model pruning was used to optimize the YOLOv4 network model. Wang Z. et al (Wang Z. et al., 2022). proposed an object detection algorithm based on YOLOv5 to achieve real-time detection of apple stem/calyx. Xie et al (Xie et al., 2023). proposed An Extremely Compressed Lightweight Model (ECLPOD) for pear object detection based on YOLOV7 to assist in the pear sorting task. These studies have shown the effectiveness of the YOLO detector. however, relatively little research has been done on pear surface defects because many high-quality pears have black spots on their surfaces, which have similar characteristics to pear surface defects and pose significant challenges to defect recognition. And there is no publicly available object detection dataset on pear surface defects. Although Xie et al (Xie et al., 2023). constructed a large dataset, they only included a single class of pear surface defects. There are no studies on detecting various types of defects that are important in the detection of pear surface defects. Given the positive developments in YOLO detectors and the pear industry, the establishment of a comprehensive benchmark for YOLO detection of surface defects in multiple classes of pears would be of significant benefit to the research community.

There are two major factors that greatly affect the detection of surface defects in pears. including the size and quality of the dataset used for model training and defect detection algorithm. Large-scale labeled image data is crucial to ensure the performance of machine vision-based algorithms. It is reported that the performance of deep learning methods on computer vision increases logarithmically with the volume of training dataset (Sun et al., 2017). In intelligent agroforestry, high-quality manually labeled datasets are still the key bottleneck to fully exploit deep learning algorithms to develop robust computer vision systems (Lu and Young, 2020). For pear surface defect detection, a good dataset should provide adequate representations of classes and specific positioning (labeled frames) of the defects, as well as labeled frames including pear contour and lighting suitable for production line, and so on. In addition to demanding domain expertise in fruit surface defect recognition preparing such a dataset is notoriously time-consuming and costly. There is no publicly available dataset for pear surface defects. some previous studies on pear surface defects have constructed their own small-scale datasets and the datasets contain only image-level annotations, so they are not suitable to detection tasks that require locating specific defect bounding boxes.

This study aims to provide the first comprehensive evaluation of the performance of state-of-the-art YOLO object detectors in multi-class pear surface defect detection, which has already been applied at Luyuan Fruit Co., Ltd. in Jianning County, Sanming City, Fujian Province. It is anticipated that this study will offer data support for the future development of machine vision-based pear quality grading systems and serve as a reference for other similar research.

## 2 Material and methodology

### 2.1 Pear surface defects dataset

**Figure 1:**
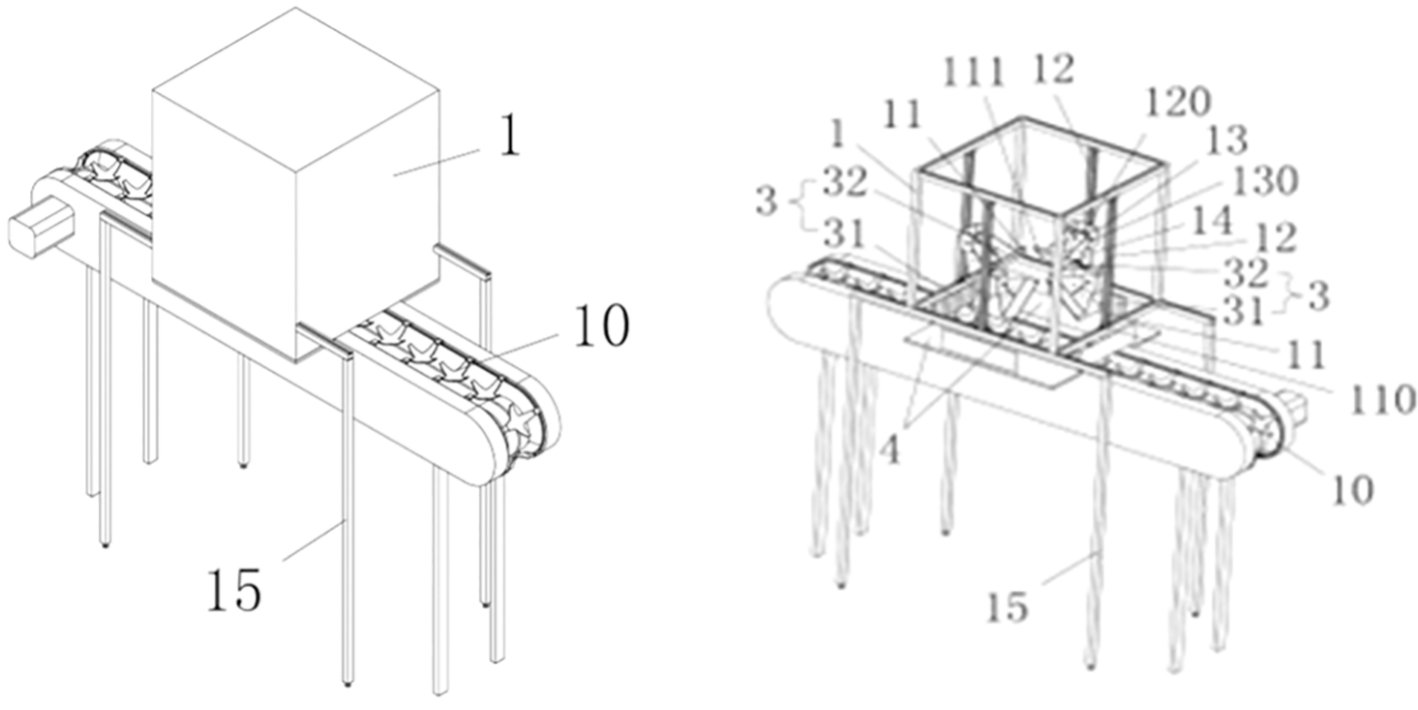
Simulation of image acquisition experimental platform, 1: Image acquisition box; 10: Fruit and vegetable conveying mechanism; 15: Support column; 4: Reflective fabric; 31: Low level lighting; 32: High level lighting; 3:Lighting device;11: Each set of cross beams; 111: Traversing guides; 12: Longitudinal beams; 120: Second bolt; 13: Camera mounting bracket; 130: Third bolt; 14: Pendulum frame.

**Figure 2:**
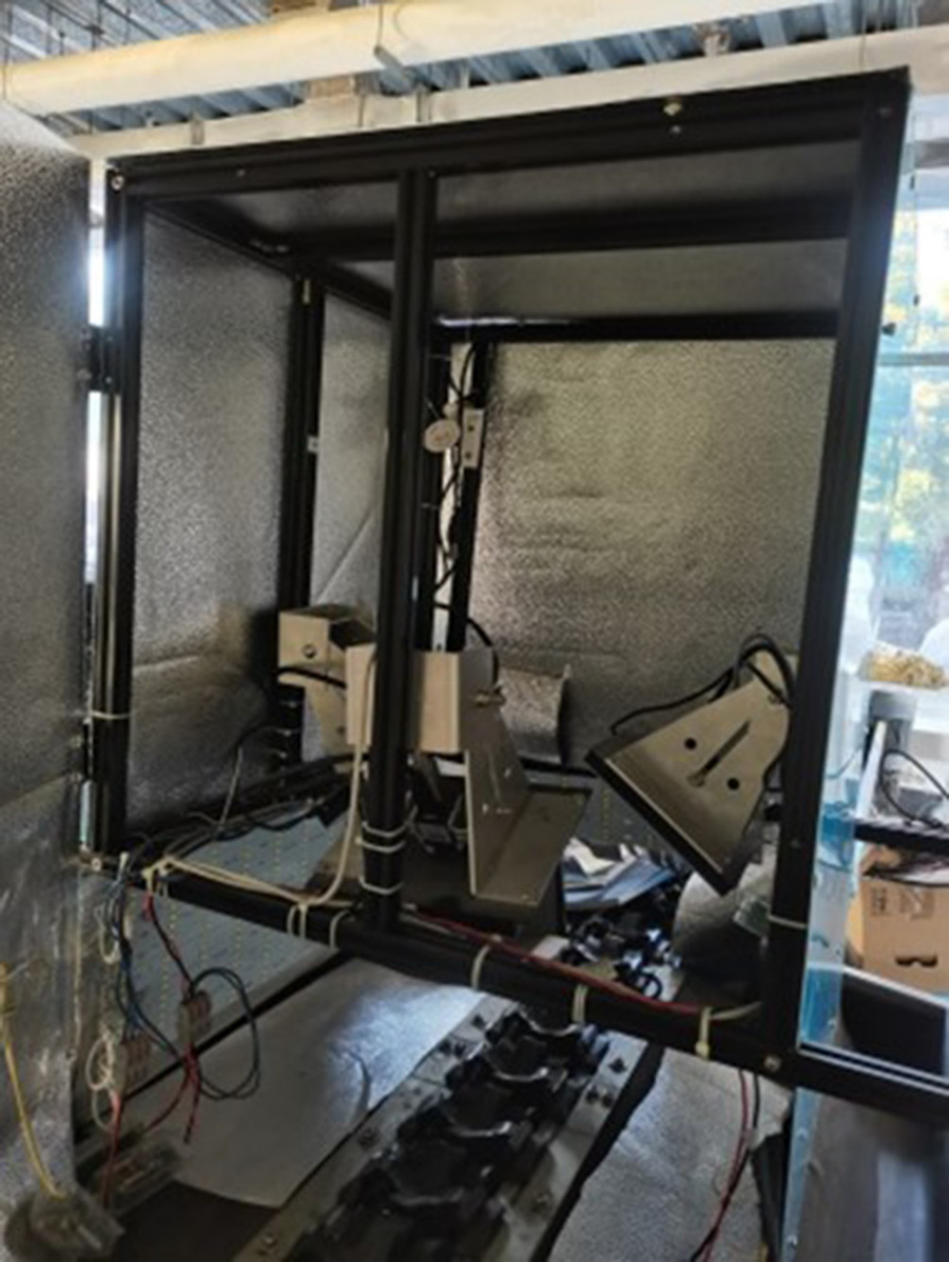
Real view of the image acquisition experimental platform.

The defect dataset in this study was collected by an image acquisition system. In order to construct the dataset, a test bed for image acquisition system was built in this study. depicted in the schematic diagram in **Figure 1**, with an interior scene photograph shown in **Figure 2**. There are mainly one TN-68A Ginon brand computer sorter, three MV-SUA1600 industrial cameras (resolution: 4608*3456, lens focal length: 6mm-18mm), one Acer Aspire V15 T5000 laptop, one flash with instantaneous flashing power of 300w (Model: CXBG-2-MC-SL-1211- 200), four 128W LED lights and other components. Image acquisition components are placed in a black dark box. In this way, each pear can be captured from three different angles by three industrial cameras. Moreover, the pears can be rolled on the conveyor belt, so that the camera directly above can captures the whole surface of the pear every 0.8s. The pears used in the experiment were pears from the orchard in Sanming City, Fujian Province. Approximately 80 boxes of various types of pears were purchased, totaling 1,000 kg. 63,000 images of pears with a resolution of 2048*1536px were captured from 27th October to 5th November, 2023, at a room temperature of 16°C on the experimental platform.

**Figure 3:**
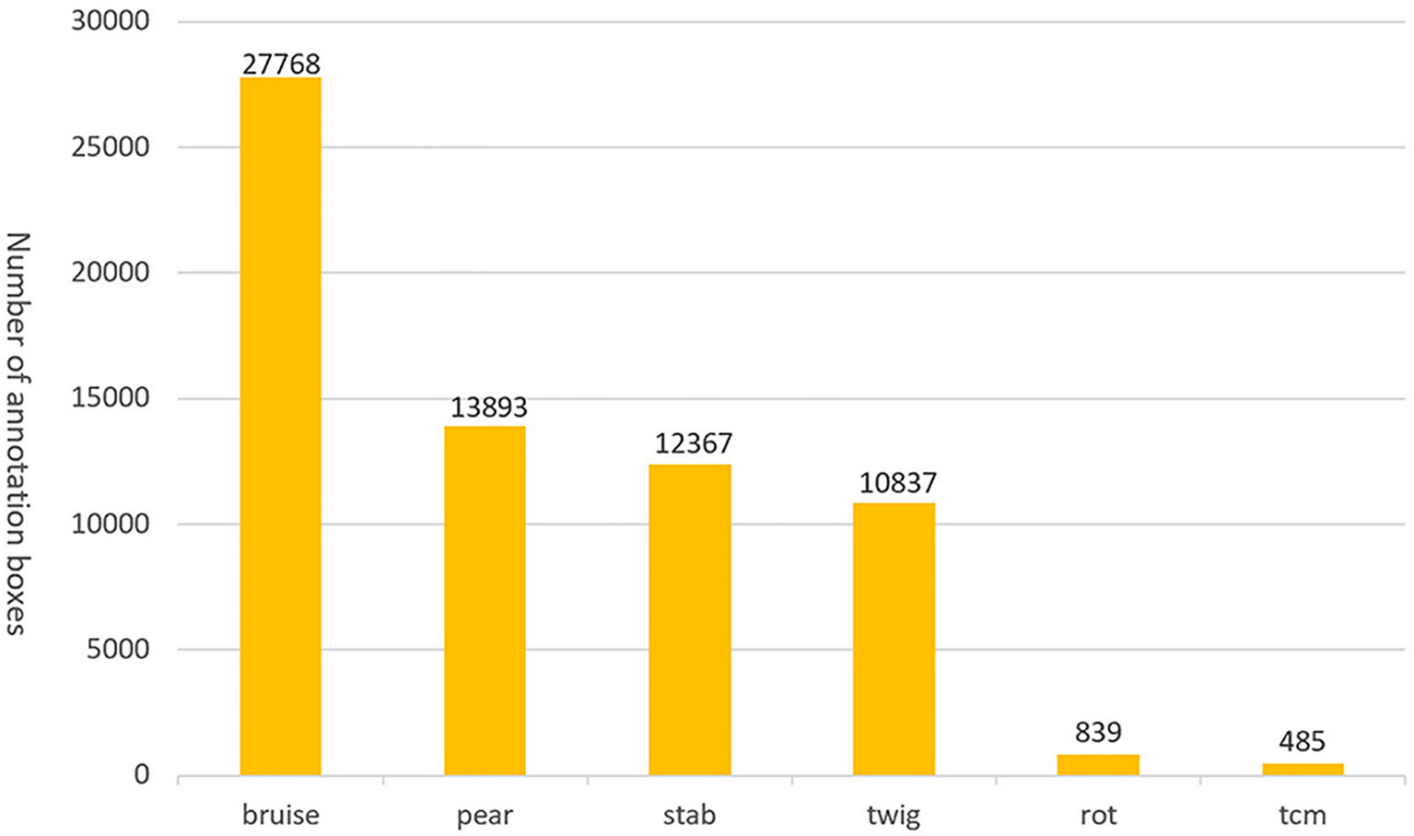
The pear surface defect detection dataset contains 13,915 images with 5 categories of defects and labels, which contains 66186 bounding boxes, due to the imbalance of defect categories and the fact that puncture wounds do not require the grading task in our study. So only 4 categories of labels were used in this.

**Figure 4:**
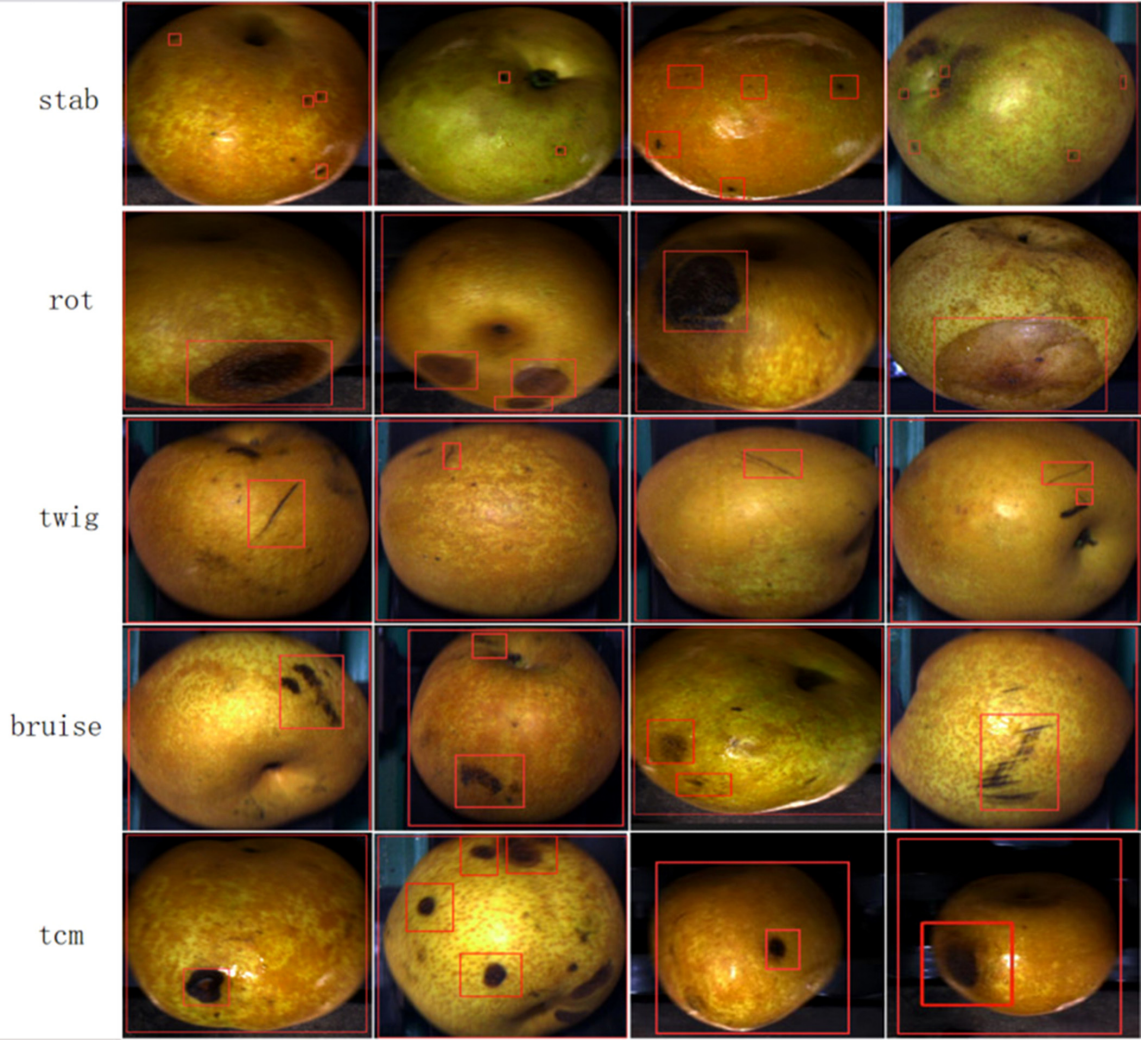
Example images of the pear surface defect dataset. Each row shows a randomly selected image of each pear surface defect, with the corresponding defect instances labelled by bounding boxes.

Since there are three different pears in a photo taken by the camera directly above, we used YOLOv3 for pear recognition, and then cropped based on the recognized candidate frames, which ensured that there is only one intact pear in each photo, and we end up with about 100,000 photos of pears. The images were labeled by trained personnel who used the Labelme labeling tool to label the bounding boxes for defects in the images. Examples of defects are labeled with their abbreviated names rather than scientific names. Where bruises and abrasions are of the same nature and extremely similar in appearance, we uniformly labeled them as mechanical injuries. The generated annotations were saved in JavaScript Object Notation (JSON) file format, and then we visualized them for multiple checks by fruit growing experts from the Chinese Academy of Agricultural Sciences in Fujian Province, China, to ensure the annotation quality. A pear surface defect dataset of 13,915 images containing 5 types of defects with 66189 annotated frames was finally obtained. Due to the unbalanced distribution of pear surface defects data, as shown in **Figure 3**, only the first 4 types of defects were considered in this study, resulting in the PearSurfaceDefects dataset, which is publicly available in the Google Cloud Drive repository. According to the needs of subsequent research and actual deployment, we will continue to update and enrich the categories of defects to improve the annotation quality of the dataset. In our experiments, we found that it is difficult for the model to distinguish those puncture wounds that have been stored for a long time. Finally, after asking experts from the Academy of Agricultural Sciences, we learned that puncture wounds can also be categorized as mechanical wounds, so we changed the labeling of puncture wounds to mechanical wounds in our experiments. To the best of our knowledge, this is the first largest public dataset in pear surface defect detection. **Figure 4** illustrates example annotated images where only one pear surface defect category is highlighted in each row of images.

### 2.2 Defect detection

#### 2.2.1 Object detection

DL-based object detectors typically consist of two main parts: the trunk and the head (Bochkovskiy et al., 2020). The backbone extracts features from high-dimensional inputs, usually pre-trained on ImageNet data, and the head predicts the class and bounding box of the object. Depending on whether or not a generative region is suggested, object detectors can be categorized as two-stage and single-stage (Liu et al., 2020). There are many layers between the backbone and the head, which are usually used to capture feature mAPs at different stages, which is also known as the neck of the object detector.

YOLOv3 (Redmon and Farhadi, 2018) achieved optimal results in real-time object detection compared to YOLO and YOLO9000 (Redmon et al., 2016; Redmon and Farhadi, 2017). YOLOv3 uses Darknet-53 as the backbone network for feature extraction, where YOLOv3-tiny is based on Darknet-19. YOLOv4 (Bochkovskiy et al., 2020) differs significantly from YOLOv3. It uses the CSPDarknet-53 backbone (Wang C. Y. et al., 2020), as well as SPP (Spatial Pyramid Pooling) and PAN (Path Aggregation Network) block necks.YOLOv4 incorporates a number of training methods, including ‘bag of freebies’ (methods that only increase the training time methods) such as CutMix (Yun et al., 2019), Mosaic Data Enhancement (Bochkovskiy et al., 2020) and DropBlock regularization (Ghiasi et al., 2018), as well as ‘Bag of freebies’ (increasing training time) and ‘bag of specials’ (slightly increasing inference time but significantly improving detection accuracy), such as mish activation (Misra, 2019), cross-stage partial connectivity and DIoU-NMS (Zheng et al., 2020). Scaled-YOLOv4 (Wang C. Y. et al., 2021a) improved YOLOv4 using cross-stage partial networks (Wang C. Y. et al., 2020), wanting to deploy YOLOv4 on a wider range of computing devices (such as regular, low-end, and high-end GPUs). Among these models, YOLOv4 for cloud GPUs- large achieves the highest detection accuracy on the COCO dataset (Wang C. Y. et al., 2021a).YOLOR (Wang C. Y. et al., 2021b) is one of the excellent YOLO target detectors, and along with other similar products such as YOLOX (Ge et al., 2021), YOLOF (Chen et al., 2021), and PPYOLOv2 (Huang et al., 2021). Based on YOLOv4-CSP (Wang C. Y. et al., 2021a), YOLOR integrates implicit and explicit knowledge to learn a generic, unified representation for multi-tasking. Tests show that YOLOR achieves comparable accuracy to Scaled-YOLOv4 in target detection tasks, but with significantly faster inference (Wang C. Y. et al., 2021b).

Shortly after the release of YOLOv4 (Bochkovskiy et al., 2020), Ultralytics LLC released YOLOv5 (Jocher, 2022), claiming that it outperforms all previous YOLO versions (Thuan, 2021). YOLOv5 only released the code and did not publish the paper, which, along with its structural similarity to YOLOv4, triggered some controversy within the computer vision community about the validity of its proposed name. The most important modification of YOLOv5 relative to YOLOv4 is the migration of the anchor frame selection process into the model (Nelson and Solawetz, 2020). Nevertheless, YOLOv5 is still widely used and in some cases performs better (Thuan, 2021). Currently, YOLOv5 is rapidly iterating in the PyTorch framework, with the latest version being YOLOv5-v7.0, which is very flexible in controlling model size and can be deployed on many devices.

The versions of YOLO following YOLOv5 (Jocher, 2022) are YOLOv6 (Li et al., 2022) and YOLOv7 (Wang C. Y. et al., 2022), both of which have demonstrated superior performance in real-time target detection. YOLOv6 features a range of updated designs in network architecture, labeling assignments, loss functions, data augmentation, and quantization, making it suitable for industrial deployments. YOLOv7, proposed by the authors of YOLOv4 (Bochkovskiy et al., 2020) and Scaled-YOLOv4 (Wang C. Y. et al., 2021a), implements an extended high-efficiency layer-aggregation network and a variety of trainable ‘free-for-all-in-a-bag’ methods (such as scheduled reparameterization and coarse-to-fine bootstrap label assignment) to enhance network training and detection accuracy without increasing inference time. On the COCO dataset, YOLOv7 outperforms previous target detectors in both speed and accuracy.

In 2023, Ultralytics LLC released YOLOv8 (Jocher, 2023), which inherits the technological strengths of the YOLO family while making significant improvements in accuracy and efficiency. Compared to its predecessor, YOLOv8 introduces an improved auto-learning anchor box mechanism and an enhanced feature extraction network, which improves detection of small objects and generalization performance in complex scenes. Additionally, YOLOv8 supports multi-scale training and inference, providing greater flexibility and adaptability. The model is published on GitHub, allowing developers to directly access and contribute to it. Although it has not undergone the traditional peer review process, it has been widely used in real-world applications demonstrating excellent performance.

While researching this work, YOLOv9 (Wang C. Y. et al., 2024) was introduced, further pushing the boundaries of the YOLO family, particularly in terms of the accuracy and efficiency of real-time object detection. It addresses the issue of information loss in deep neural networks by introducing Programmed Gradient Information (PGI) and reversible functions, technical innovations that significantly improve the model’s ability to learn and retain critical information.YOLOv9 also employs the Generalized Efficient Layer Aggregation Network (GELAN), which improves parameter utilization and computational efficiency. Compared to the previous generation of models, YOLOv9 achieves higher detection accuracy and speed while maintaining or reducing computational requirements, making it ideal for high-performance real-time applications. The release of YOLOv9 is also available on GitHub for easy access and optimization by the community, although it has not undergone the traditional academic review process.

In the field of object detection, the application of Transformer (Vaswani et al., 2017) models has increasingly garnered attention. The DETR (Detection Transformer) (Carion et al., 2020) model integrates the strengths of traditional CNNs and Transformer models, while RT-DETR (Zhao et al., 2024) employs CNN as the backbone for feature extraction and incorporates a Transformer encoder in the final layer of the feature extraction network to establish global feature correlations. In the neck network, RT-DETR utilizes a CCFM module, inspired by the PAFPN structure, and integrates top-down and bottom-up feature maps through a fusion module, further enhancing the model’s performance in multi-scale object detection. The recently released RT-DETRv2 (Lv et al., 2024) improves upon RT-DETR by introducing multi-scale selective feature extraction, optional discrete sampling operators, and optimized training strategies, further enhancing detection performance and practicality while maintaining efficient real-time processing. In contrast, D-FINE (Peng et al., 2024) is a state-of-the-art real-time object detection model that significantly enhances localization accuracy and overall performance by redefining the bounding box regression task within the DETR model. It combines fine-grained distribution refinement (FDR) with globally optimal localization self-distillation (GO-LSD), achieving an impressive balance between speed and accuracy.

In this study, the seven YOLO object detectors described above, including Scaled-YOLOv4, YOLOR, YOLOv5, YOLOv6, YOLOv7, YOLOv8, and YOLOv9 were selected for use in developing a surface defect detection model for the pear dataset. Additionally, three Transformer-based object detectors—RT-DETR, RT-DETRv2, and D-FINE—were chosen for comparison. Although previous studies had conducted pear defect identification, they were based on classification models and were not able to pinpoint the location of the defects (Sun et al., 2022; Zhang et al., 2021). There is a recent study that used a detection model to identify pear surface defects, but it only identified one pear surface defect (Xie et al., 2023). A comprehensive evaluation study of a range of YOLO detectors detecting multiple categories of surface defects for the Mile Pear dataset species has not yet been conducted. It is important to note that the selected detectors come with open-source software packages provided by their developers, with hyperparameters for training summarized in **Table 1**. These software packages were adapted for use in this study to train the pear surface defect detection model.

**Table 1:** Summary of benchmark models and hyperparameters.

Index	Yolo models	Total Batch	Epochs	Optimizer	Momentum	Weight Decay	Learning Rate (lr0)	Scheduler	Input Size	Reference
**1**	Scaled-YOLOv4	120	100	SGD	0.937	0.0005	0.0100	Cosine Annealing		Wang C. Y. et al. (2021a)
**2**	YOLOR	120	100	SGD	0.937	0.0005	0.0100	Cosine Annealing		Wang C. Y. et al. (2021b)
**3**	YOLOv5	120	100	SGD	0.937	0.0005	0.0100	Cosine Annealing		Jocher (2022)
**4**	YOLOv6	120	100	SGD	0.937	0.0005	0.0100	Cosine Annealing		Li et al. (2022)
**5**	YOLOv7	120	100	SGD	0.937	0.0005	0.0100	Cosine Annealing		Wang C. Y. et al. (2023)
**6**	YOLOv8	120	100	SGD	0.937	0.0005	0.0100	Cosine Annealing		Jocher (2023)
**7**	YOLOv9	120	100	SGD	0.937	0.0005	0.0100	Cosine Annealing		Wang C. Y. et al. (2024)
**8**	RT-DETR	120	100	AdamW	N/A	0.0000	0.0001	Cosine Annealing		Zhao et al. (2024)
**9**	RT-DETRv2	120	100	AdamW	N/A	0.0000	0.0001	Cosine Annealing		Lv et al. (2024)
**10**	D-FINE	120	100	AdamW	N/A	0.0001	0.0008	Cosine Annealing		Peng et al. (2024)

#### 2.2.2 Experiments

**Figure 5:**
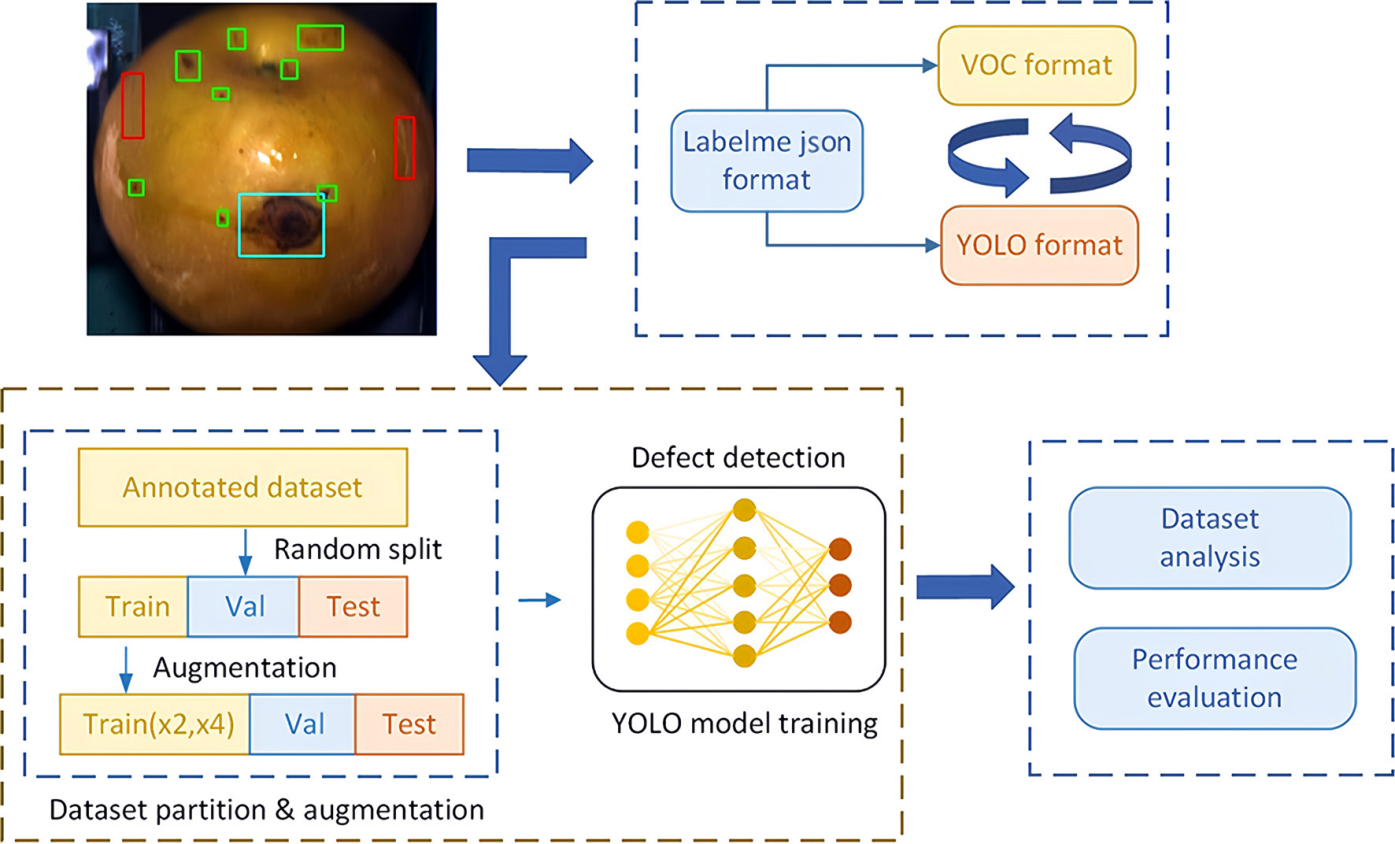
Flow of YOLO object detector for pear surface defect detection.

**Figure 5** illustrates a flowchart of the modeling used for pear surface defect detection. The original image annotations in JSON format, generated using the labelme annotation tool were converted into YOLO format labels for YOLOv4, YOLOv5, YOLOv6, YOLOv7, YOLOv8 and YOLOv9. After the format conversion, the image dataset was randomly divided into training, validation, and test subsets according to a division ratio of 8:1:1 (11,132-1,391-1,392 images), respectively.

To facilitate the training of the model, the selected YOLO object detectors were trained using migration learning (Zhuang et al., 2020) by fine-tuning the pre-training weights (Lin et al., 2014). During training, the image of the input model was adjusted to a resolution of 640 × 640 pixels, as shown in the YOLO network architecture.YOLOv5 through YOLOv9 were implemented in the PyTorch framework (version 2.0.1 + cu118) (Paszke et al., 2019), while Scaled-YOLOv4 and YOLOR were in PyTorch (version 1.8.2 + cu111). RT-DETR, RT-DETRv2, and D-FINE were implemented in the PyTorch framework (version 2.0.1 + cu118). All models were trained with a total batch size of 120 for 100 epochs. We trained all detectors on 8 GPUs. All detectors using cosine annealing (He et al., 2019) to adjust the learning rate over time. All other hyperparameters used the default settings from the official implementation (see **Table 1**). The computational resources for the model training and testing experiments are composed of three GPU compute servers, each with the following GPU computational resources: 2 AMD EPYC 7763 CPUs with 128 cores. 16 sticks of 64GB DDR4 3200MHz RAM for a total of 1TB of RAM; 2 blocks of 3.84TB U.2 2.5-inch PCIE 4.0 enterprise-grade SSDs; 1 dual-port 10 Gigabit fiber optic network card (Full optical module); 8 NVIDIA 3090 turbo cards.

**Figure 6:**
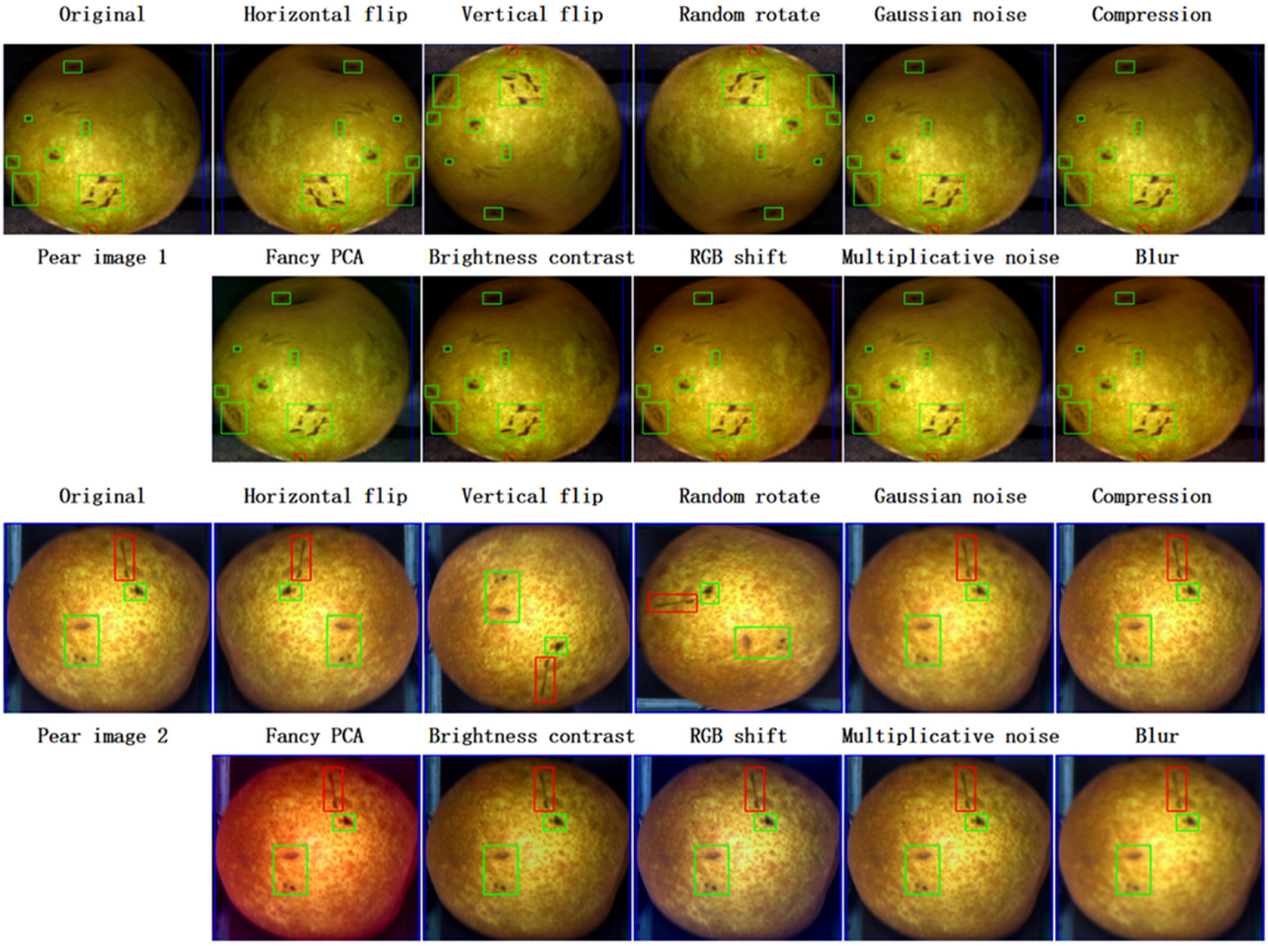
Example of basic image enhancement of two pear defect images, each processed by ten different conventional image enhancement methods. Images with different colored bounding boxes indicate different defect types and the bounding box information is preserved during the image enhancement process.

In image classification, data enhancement can improve model accuracy in object detection (Zoph et al., 2020). Although, YOLO introduces data augmentation during model training, data augmentation used in isolation can still improve model accuracy. Therefore, in order to improve the detection accuracy, the training dataset is augmented by randomly applying one of the 10 geometric and photometric transformations shown in **Figure 6** on the individual training images. Here, to preserve the computational cost, only two cases of data augmentation are considered, i.e., doubling and quadrupling the original training set, respectively (**Figure 5**). These transformations are implemented using the image enhancement package Albumentations (Buslaev et al., 2020). Bounding box information for each defect also needs to be saved during data enhancement. In this study, Data enhancement experiments were conducted on three YOLO models, namely, YOLOv6n (as shown in **Table 2**, with the lowest value at mAP@0.5), YOLOv4-p7 (as shown in **Table 2**, with the highest value at mAP@0.5) and YOLOv5s, to check their effectiveness.

**Table 2:** Pear surface defect detection performance of 27 YOLO detectors on the test dataset.

Index	YOLO models		Precision	Recall	mAP@0.5	mAP@[0.5:0.95]	Inference Time(ms)
1	Scaled-YOLOv4	YOLOv4-P5	58.60	76.00	67.20	45.40	14.0
2		YOLOv4-P6	57.50	80.60	69.60	46.00	15.8
3		YOLOv4-P7	56.20	80.40	71.20	46.50	29.0
		Average	57.43	79.00	69.33	45.97	19.6
4	YOLOR	YOLOR-P6	59.30	74.20	67.20	43.70	3.9
5		YOLOR-w6	58.70	73.90	68.90	45.60	4.6
6		YOLOR-CSP-X	56.80	75.30	69.10	44.30	10.6
		Average	58.27	74.47	68.40	44.53	6.4
7	YOLOV5	YOLOV5n	67.50	65.90	65.30	44.30	1.2
8		YOLOV5s	71.60	65.10	65.40	43.60	1.6
9		YOLOV5m	70.90	65.40	64.50	43.60	3.5
10		YOLOV5l	68.40	62.90	64.30	43.20	5.8
11		YOLOV5x	68.80	63.20	65.40	43.20	11.0
		Average	69.44	65.30	64.98	43.58	4.6
12	YOLOv6	YOLOV6n	68.20	63.80	63.60	44.30	1.1
13		YOLOV6s	66.80	59.00	63.70	44.00	2.3
14		YOLOV6m	65.10	60.00	64.70	44.70	4.8
15		YOLOV6l	65.20	60.50	63.80	45.10	7.4
		Average	66.33	60.83	63.95	44.53	3.9
16	YOLOv7	YOLOv7	70.50	63.80	66.50	44.30	4.0
17		YOLOv7x	68.20	65.40	67.30	44.40	6.4
28		YOLOv7W6	68.30	65.20	65.90	44.00	3.8
19		YOLOv7E6	69.00	61.30	64.10	43.80	5.6
20		YOLOv7D6	67.30	63.50	64.20	42.50	6.8
		Average	68.66	63.84	65.60	44.94	5.3
21	YOLOv8	YOLOV8n	68.90	62.10	64.80	44.10	1.6
22		YOLOV8s	67.10	63.50	64.80	44.50	2.7
23		YOLOV8m	67.10	65.40	65.30	44.50	5.2
24		YOLOV8l	69.00	64.00	64.90	45.10	8.1
25		YOLOV8x	68.20	63.80	65.60	44.30	13.4
		Average	68.06	63.76	65.08	44.70	6.2
26	YOLOv9	YOLOV9-c	69.60	65.10	68.30	45.60	14.0
27		YOLOV9-e	72.40	67.20	70.20	47.10	18.1
		Average	71.00	66.15	69.25	46.35	16.05

Percentages of precision, recall, mean average precision (mAP) and inference time.

### 2.3 Performance evaluation indicators

The performance of the object detector in pear surface defect detection is evaluated based on detection accuracy, model complexity, and inference time (Dang et al., 2022), as described below.

#### 2.3.1 Detection accuracy

The detection accuracy of the trained model was evaluated on the test data using common metrics for object detection (Everingham et al., 2015; Padilla et al., 2020), which include precision (P), recall (R), and mean average precision (mAP, i.e., mAP@0.5 and mAP@[0.5:0.95]). Among these metrics, mAP is the main metric used to evaluate object detectors in multi-category object detection.

#### 2.3.2 Number of model parameters

The number of model parameters is a direct indicator for assessing the complexity of a model and is one of the key factors that must be taken into account when actually deploying it. Models with more parameters usually require more memory, which affects their deployability on various devices. In addition, the number of parameters significantly affects computational cost and inference time. More parameters mean more computational resources and longer processing time are required in real-world applications, which may affect the efficiency and usefulness of the model. (see Section 2.3.3 for more details).

#### 2.3.3 Computational cost and inference time

Floating point operations (FLOPs) are commonly used as a measure of the computational power of a model, i.e. the number of operations required to run the model to process a single instance. as another criterion for evaluating model complexity. Inference time, which refers to the time taken by the model to make predictions about the input image, is critical for real-time applications. Here, the inference time for each YOLO detector is the average time required to compute predictions for all test set images. Unlike FLOPs, inference time is affected by the computational hardware, so the same model may have different inference times on different hardware. FLOPs provide a hardware-independent measure of computational complexity, whereas inference time reflects performance in real-world deployments.

## 3 Results and discussion

### 3.1 Performance of the YOLO model

**Figure 7:**
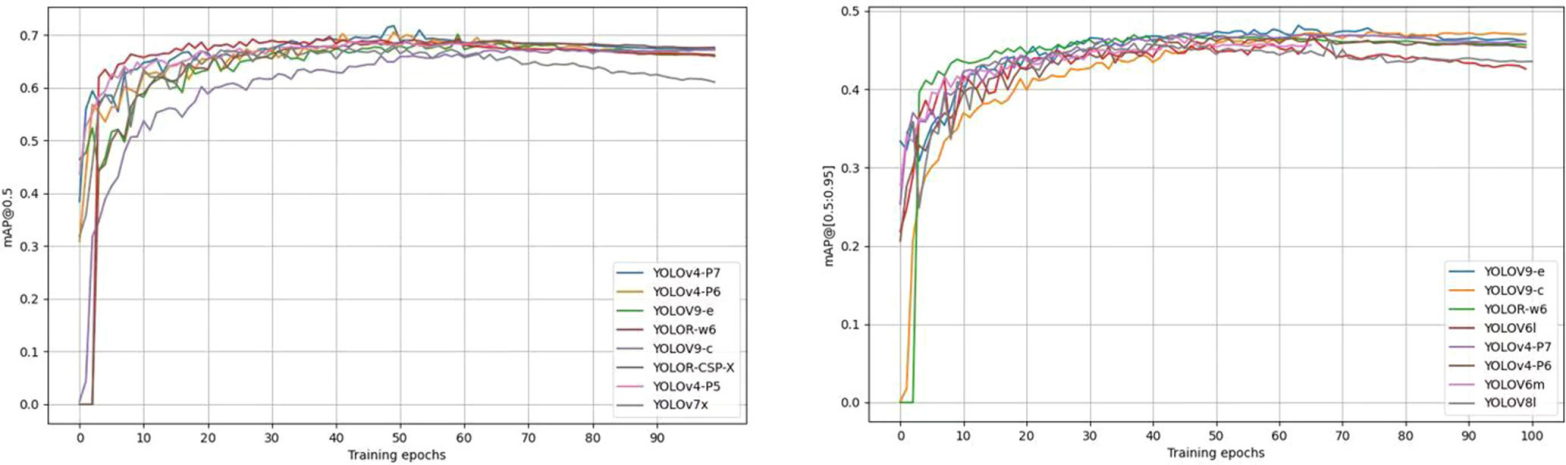
Training curves of mAP@0.5 and mAP@[0.5:0.95] of the YOLO model for the first 8 defect detections.

**Figure 7** shows the training curves for mAP@0.5 and mAP@[0.5:0.95] for the top 8 detectors in terms of accuracy of pear surface defect detection. The training curves for the other models are not shown here due to space constraints. Overall, these models show objective training performance in terms of fast convergence and high detection accuracy, achieving 63% of mAP@0.5 and 45% of mAP@[0.5:0.95] within 50 training cycles. Training showed that the accuracy of all models (including those not shown in **Figure 7**) stabilized after more than 70 cycles, validating the adequacy of 100 cycles of training in this study.

**Figure 8:**
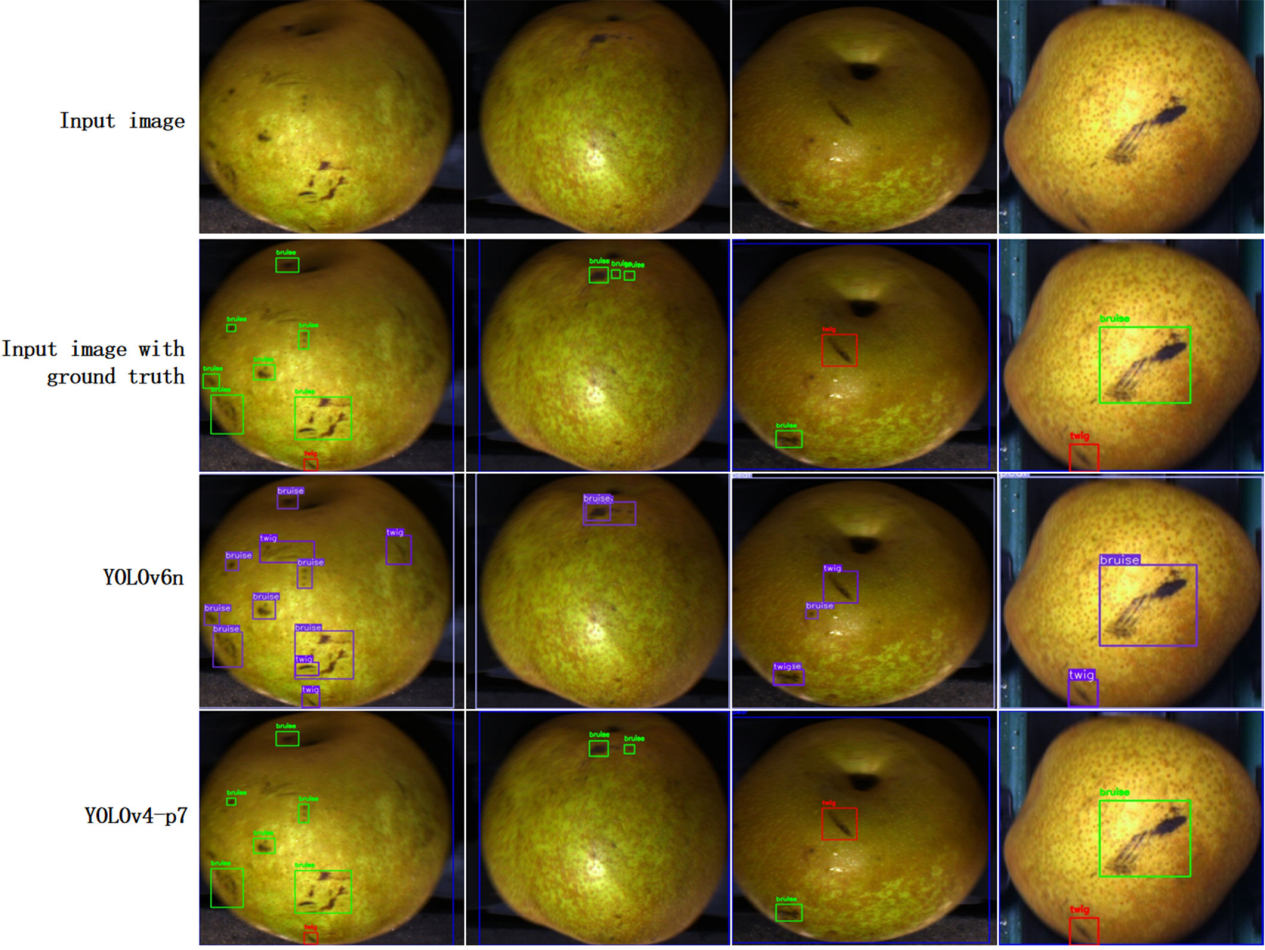
Pear surface defect detection with prediction boxes.

**Table 2** summarizes the performance results of all YOLO detection models. The trained models are publicly available on the project’s GitHub site. Overall, all 27 models achieve considerable accuracy in detecting defects on pear surfaces. mAP@0.5 values range from 63.60% for YOLOv6n to 71.20% for Scaled-YOLOv4-p5. The accuracy of mAP@[0.5:0.95] ranges from 42.50% for YOLOv7D6 to 47.10% for YOLOv9-e. mAP@[0.5:0.95] values are lower than those of mAP@0.5 because the former uses higher IoU thresholds (which implies stricter criteria) to compute the APs. **Figure 8** illustrates the results obtained by the YOLOv6n and Scaled -YOLOv4-p5 predicted image examples. These two models produce visually good predictions in the pear surface defect complication images, even for defects that are difficult to distinguish by the human eye. The prediction results for the test images have also been collated into a video presentation, which can be viewed on the GitHub website. Overall, these results demonstrate the effectiveness of the chosen YOLO object detector in multi-class surface defect detection.

In order to examine the performance differences between the seven different types of YOLO detectors, an average accuracy value was obtained by calculating the average accuracy of the results for the different model variants within each detector type (see **Table 2**). On average, Scaled-YOLOv4’s mAP@0.5 and YOLOv9’s mAP@[0.5:0.95] were the best, with 70.39% and 47.75%, respectively, followed by YOLOv9 and YOLOR’s mAP@0.5, with similar and higher accuracies, and then YOLOv7, YOLOv5, YOLOv6 and YOLOv8 While the average accuracies may not be a rigorous metric for comparing different types of YOLO detectors, their differences seem to indicate that the YOLOv4-based detection models (models indexed 1-6 in **Table 2**) perform better than the YOLOv5- and YOLOv6-based models in terms of pear surface defect detection accuracy. It is worth noting that more recent YOLO detectors (YOLOv8 and YOLOv9) achieved the most recent accuracies when benchmarked on the COCO dataset, but they did not outperform the earlier YOLO detectors, including Scaled-YOLOv4 and YOLOR. The exact reasons for the lower accuracy of these two newer models are yet to be investigated, but it is important to note that the performance of YOLO detectors may be specific to the object detection task and dataset, as observed in the literature (Nepal and Eslamiat, 2022) and also discussed at https://github.com/AlexeyAB/darknet/issues/5920. The *ad hoc* design of network training and data enhancement techniques in recent YOLO detectors may not lead to performance improvements when transferring YOLO models pre-trained and evaluated on COCO data to a different domain dataset, unless dedicated domain adaptation work has been performed (Dang et al., 2023).

The detection accuracy of each pear surface defect category was further examined. Due to space limitations, only the results obtained by YOLOv5s and YOLOv6s are shown here, summarized in **Table 3**. The accuracy of pear surface defect detection may be affected by factors such as the number and size of bounding boxes, biological variation between classes, and defect similarity between classes. During annotation, we found that abrasions and bruises were very similar, and we eventually referred to them as mechanical injuries. Moreover, punctures and mechanical injuries already present in the dataset are also very similar, with many defects have very similar features, making it very difficult to distinguish them. For the characteristic class such as pear, each detector achieved a mAP value of 99%, but for defects with complex features, the accuracy was not very high, which determined our direction for future maintenance and expansion of this dataset.

**Table 3:** The detection results of YOLOV5n and YOLOV6n for each category, where P, R and mAP represent the precision, recall and average precision respectively.

Index	Class	YOLOv5n				YOLOv6n			
		P	R	mAP@0.5	mAP@[0.5:0.95]	P	R	mAP@0.5	mAP@[0.5:0.95]
**1**	**all**	**67.50**	**65.90**	**65.30**	**44.30**	**68.20**	**63.80**	**63.60**	**44.30**
**2**	**pear**	**99.40**	**99.70**	**99.40**	**96.00**	**99.20**	**99.40**	**99.20**	**97.80**
**3**	**bruise**	**53.5**	**53.20**	**50.00**	**18.80**	**51.80**	**49.80**	**48.70**	**20.20**
**4**	**twig**	**52.60**	**46.40**	**44.10**	**17.00**	**51.30**	**49.80**	**48.70**	**20.00**
**5**	**rot**	**64.40**	**64.30**	**67.50**	**45.50**	**70.30**	**57.10**	**58.50**	**39.30**

**Figure 9:**
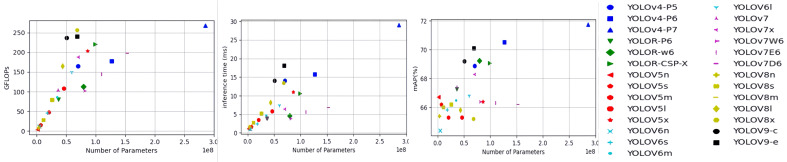
GFLOPs and inference time (milliseconds) versus number of parameters (millions), and mAP@[0.5:0.9] versus inference time. Detection models in the same type of YOLO detector are labeled with the same color markers. GFLOPs stands for 100 billion floating point operations (FPLOPs), which is equal to 10^9 FLOPs, and mAP stands for mean average precision.

The first two sub-figures in **Figure 9** depict the relationship between the number of model parameters and GFLOPs and inference time for all tested YOLO detectors. GFLOPs and inference time increase linearly with the number of model parameters. Among the seven types of YOLO models, Scaled-YOLOv4 appears to have the highest GFLOPs and the longest inference time of 29.00 milliseconds. In contrast, the YOLOv5 model, especially the two simplified versions YOLOv5n and YOLOv5s, is the most computationally efficient and has fastest inference (consuming less than 2 milliseconds). **Figure 9** also depicts the relationship between model inference time and mAP@[0.5:0.95]. There may be a trade-off between accuracy and inference time in model selection, as high accuracy is often associated with longer inference time. Overall, all YOLO detectors tested in this study have the potential to perform surface defect detection under real-time conditions of tens or even hundreds of frames per second. However, since the models were tested on advanced computing hardware, further testing of these models on embedded devices is needed.

### 3.2 Data augmentation

**Figure 10:**
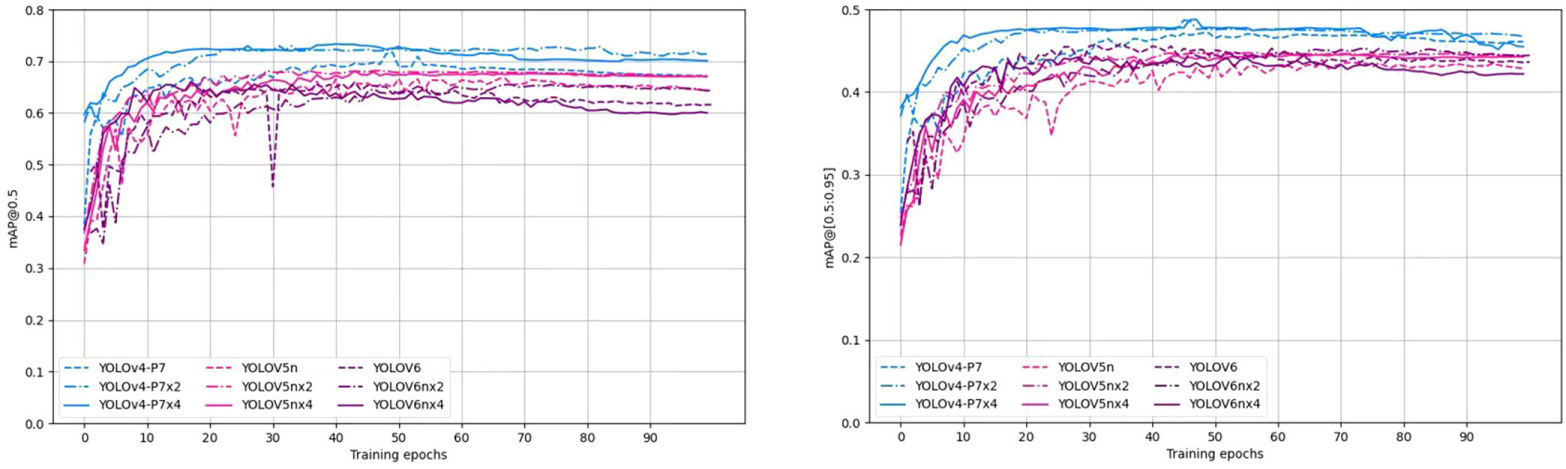
Training accuracy curves of three YOLO models with and without data augmentation.

To investigate the impact of the data augmentation methods described in Section 2.2.2 on the model performance, experiments were conducted on three selected YOLO detectors, namely YOLOv6n (worst performance in terms of mAP@0.5), YOLOv4-P7 (best performance in terms of mAP@0.5), and YOLOv5s, with and without data augmentation. **Figure 10** shows the training curves of the three YOLO detectors. Obviously, data augmentation significantly speeds up the training process for all three models. Both YOLOv4-P7 and YOLOv5n achieve performance gains in terms of mAP@0.5 and mAP@[0.5:0.95]. **Table 4** shows the comparison of the detection accuracy metrics of the YOLO detectors with and without data augmentation. Data augmentation has a positive impact on the performance of YOLOv4-P7 and YOLOv5n. Using four times the data augmentation, YOLOv4-P7 achieves significant increases in mAP@0.5 and mAP@[0.5:0.95], from 71.20% to 73.20% for mAP@0.5 and from 46.50% to 47.80% for mAP@[0.5:0.95]. YOLOv5n also achieves slight increases in mAP@0.5 and mAP@[0.5:0.95], despite the inclusion of advanced data augmentation (CutMix and Mosaic data augmentation) in its modeling process. However, for YOLOv6n, mixed performance is observed, indicating that the data augmentation proposed in Section 2.2.2 may not be effective enough for YOLOv6n. Considering that the YOLOv6 detector already incorporates standard data augmentation methods, further non-customized data augmentation may not necessarily contribute to the accuracy improvement. Further research is needed to optimize the data augmentation strategy for the YOLO detector for pear surface defect detection.

**Table 4:** Defect detection performance of three YOLO models on the pear surface defect detection dataset (without data augmentation and with data augmentation).

Models	Precision	Recall	mAP@0.5	mAP@[0.5:0.95]
YOLOV6n	68.20	63.80	63.60	44.30
YOLOV6n-agu(X2)	62.40	64.00	64.00	44.80
YOLOV6n-agu(X4)	64.20	65.10	63.10	44.90
YOLOv4-P7	56.22	80.44	71.20	46.50
YOLOv4-P7-agu(X2)	55.60	79.40	73.10	47.60
YOLOv4-P7-agu(X4)	57.10	78.80	73.20	47.80
YOLOV5n	67.50	65.90	65.30	44.30
YOLOV5n -agu(X2)	66.30	66.50	67.10	44.80
YOLOV5n-agu(X4)	67.10	65.37	67.10	44.10

### 3.3 Discussion

Research on multi-class pear surface defect detection and localization is scarce in the literature. This is partly due to the lack of suitable pear surface defect datasets with multi-class bounding box annotations. As machine vision-based fruit grading systems are evolving towards the next generation of high-accuracy and control for specific pear defects, it becomes increasingly important to distinguish the species of pear surface defects and detect individual defect instances. Although considerable efforts have been made in the classification of pear image defects (Sun et al., 2022; Zhang et al., 2021), few studies have focused on multi-class pear surface defect detection, especially with large-scale, multi-class pear surface defect data. Extending from our previous work on ‘Cuiguan Pear’ grading (Liu et al., 2023), this study addresses this issue by creating and releasing a 6-class pear surface defect dataset with tens of thousands of bounding box annotations collected on an image acquisition system. In addition, we establish a comprehensive benchmark for pear surface defect detection using the state-of-the-art YOLO detector, which performs well in terms of detection accuracy and fast inference time.

In addition to YOLO models, we further explored Transformer-based object detection models, including RT-DETR, RT-DETRv2, and D-FINE. These models demonstrated significant potential in handling complex pear surface defect images. **Table 5** presents a comparison between the fastest inference and highest mAP@0.5 YOLO detection models and advanced Transformer-based object detection models. For the Transformer models, we selected both the smallest and largest parameter versions for experimentation. Among these Transformer-based models, RT-DETRv2-X achieved the highest precision with an mAP@0.5 of 68.10%, approaching the performance of YOLOv4-P7, albeit with an inference time of 31.5 ms, which is slightly slower than the latter. D-FINE-N achieved the fastest inference time among the Transformer models at 10.0 ms, but both its precision (mAP@0.5) and inference speed were inferior to YOLOv6. Based on the experimental results, YOLO models appear to be better suited for pear surface defect detection and grading tasks, while Transformer-based models exhibit remarkable potential in balancing precision and inference time.

**Table 5:** Performance of YOLO and transformer-based models in pear surface defect detection on the test dataset, detailing precision, recall, mean average precision (mAP), and inference time.

Index	YOLO models		Precision	Recall	mAP@0.5	mAP@[0.5:0.95]	Inference Time(ms)
1	YOLOv6	YOLOV6n	68.20	63.80	63.60	44.30	1.1
2	Scaled-YOLOv4	YOLOv4-P7	56.22	80.44	71.20	46.50	29.0
3	RT-DETR	rtdetr_r18vd	65.30	67.90	61.70	41.50	19.6
4		rtdetr_r101vd	71.10	65.10	68.00	46.40	33.0
5	RT-DETRv2	RT-DETRv2-S	68.20	63.80	62.10	41.50	18.0
6		RT-DETRv2-X	71.00	65.30	68.10	46.50	31.5
7	D-FINE	D-FINE-N	68.20	63.80	62.70	42.10	10.0
8		D-FINE-X	66.80	59.00	65.20	43.40	18.8

This study has several limitations that require further improvements. This study does not aim to evaluate all YOLO-based object detectors for pear surface defect detection, there are other YOLO models with good performance, such as YOLOX (Ge et al., 2021), PP-YOLOv2 (Huang et al., 2021), and YOLOv3 (Redmon and Farhadi, 2018), were not experimented,. However, these models will be tested and incorporated in the benchmark that we will actively update as we continue to update and expand the pear surface defect detection dataset with future efforts. The data augmentation experiments in this study need to be improved in a more systematic way. Cross-dataset evaluation is required to assess the robustness or generalization ability of data augmentation methods. Critical factors that may affect model performance, such as the number of augmented images and image augmentation or synthesis approaches, should be further studied. This will be of great benefit in reducing the amount of effort and resources required for image acquisition and annotation. In this regard, it is worth noting that small object data augmentation methods may be more suitable for the pear surface defect dataset (most defects are small objects), because small object data augmentation methods are a type of data augmentation method designed for detecting or recognizing small objects (such as small objects or low-resolution objects in images). When dealing with small objects, there are usually some challenges, such as small object size, low information content, and susceptibility to noise interference. Therefore, small object data augmentation (Kisantal et al., 2019) aims to improve the performance and robustness of small object detection or recognition models through a series of technologies.

In future research, the pear surface defect detection model developed in this study will be systematically verified by being deployed on the pear grading equipment built by the Fujian Academy of Agricultural Sciences. The focus will be on confirming the model’s out-of-distribution robustness (Hendrycks et al., 2021), which is crucial for practical applications. Preliminary tests on the fruit grading equipment using the YOLOv4-p7 model achieved the best mAP@0.5 in this study, which confirmed its effectiveness in the fruit grading equipment of Fujian Academy of Agricultural Sciences. Accurate grading of pears was achieved. However, more extensive and dedicated research is needed to understand and improve the robustness under different imaging conditions.

The pear defect dataset and software program developed in this research will be available and public gradually. It is expected that this research will have a positive impact on future research to develop machine vision-based surface defect detection for pears (and possibly other fruits). The main contributions of this paper are as follows:

1. A diverse dataset of pear surface defects including 13,915 images in 6 categories.
2. A comprehensive evaluation and benchmark of 27 state-of-the-art YOLO object detectors for multi-class surface defect detection.
3. The effect of data augmentation on the performance of the YOLO model was examined.

## 4 Conclusion

Pear surface defect detection is crucial for pear quality grading. The detection of pear surface defects involves constructing a large-scale, accurately labeled defect dataset and thereby developing supervised learning models. This paper introduces the largest pear surface defect detection dataset related to the pear quality grading system to date, including 13,915 images in 6 categories of surface defect with a total of 66,189 bounding box annotations, which were collected on an image acquisition system built. A comprehensive benchmark suite of 27 selected YOLO object detectors was established through transfer learning for multi-class pear surface defect detection, which was evaluated in terms of detection accuracy, model complexity, and inference time. The inference time of YOLOv6n is just 1.1 milliseconds, enabling real-time pear surface defect detection. Even the slower YOLOv4-P7 achieves an inference time of only 29.0 milliseconds, further demonstrating that these YOLO models are fully capable of performing real-time pear surface defect detection. Having captured a vast collection of over 100,000 photos, we have so far annotated only a fraction of these, just above 10,000. Nonetheless, our team will continue to update and maintain the dataset to advance our detection capabilities.

## Data Availability

The data set and software programs used in this study are publicly available at https://drive.google.com/drive/folders/1INRSHXMOqBf39mobRi6iKkm01JjMyVgK and https://github.com/junshengchen/PSD.
